# Characterization of *Photobacterium damselae* subsp. *damselae* isolated from a spotted seal (*Phoca largha*) (Pinnipedia: Phocidae) stranded in Korea

**DOI:** 10.3389/fvets.2025.1574705

**Published:** 2025-08-07

**Authors:** Tae Seon Cha, Seon Young Park, Kyunglee Lee, Eun Jeong Park, Jong Beom Na, Ye Bin Kim, Keeman Lee, Soojin Lim, Namgyu Uh, Ji-Youl Jung, Byung Yeop Kim, Bumkeun Kim, Jee Eun Han, Ji Hyung Kim

**Affiliations:** ^1^Department of Food Science and Biotechnology, College of Bionano Technology, Gachon University, Seongnam, Republic of Korea; ^2^Veterinary Drugs and Biologics Division, Animal and Plant Quarantine Agency, Gimcheon, Republic of Korea; ^3^Cetacean Research Institute, National Institute of Fisheries Science, Ulsan, Republic of Korea; ^4^College of Veterinary Medicine and Veterinary Medical Research Institute, Jeju National University, Jeju, Republic of Korea; ^5^Department of Marine Industrial and Maritime Police, College of Ocean Science, Jeju National University, Jeju, Republic of Korea; ^6^Institute for Veterinary Biomedical Science, Department of Veterinary Medicine, Kyungpook National University, Daegu, Republic of Korea

**Keywords:** *Photobacterium damselae* subsp. *damselae*, marine mammal, risk factor, cytotoxicity, histamine poisoning

## Abstract

**Introduction:**

*Photobacterium damselae* subsp. *damselae* (PDD) is an emerging marine bacterial pathogen that infects marine animals and humans, causing fatal necrotizing fasciitis and histamine fish poisoning. Despite its clinical and ecological importance, the microbiological and genomic characteristics of PDD remain largely unknown.

**Methods:**

We report the first case of systemic infection caused by PDD in a free-ranging spotted seal (*Phoca largha*) stranded in Korea. Histopathological and microbial examinations were performed, followed by genomic analysis of the isolated PDD strain GCUPdd. Histamine production capability and cytotoxic effects on human cells were also evaluated.

**Results:**

PDD was identified as the presumptive cause of systemic infection in the seal. Genomic analysis revealed the presence of pPHDD-like plasmid and major virulence factors including damselysin, phobalysin, and phospholipase. Strain GCUPdd harbored a gene cluster for histamine production (histidyl-tRNA synthetase, histidine decarboxylase, and histidine-histamine antiporter) and exhibited significantly higher histamine-producing ability than the reference PDD strain. The strain also demonstrated cytotoxic effects on human cells.

**Discussion:**

Although the pathogenic role of PDD in pinnipeds remains unclear, this study highlights its zoonotic potential and the importance of monitoring PDD in marine environments. Our findings contribute to understanding risk factors for histamine fish poisoning and provide insights into microbial diversity in marine mammals, emphasizing the need for further surveillance concerning PDD pathogenicity and role in public health.

## 1 Introduction

The genus *Photobacterium* (*P*.) is one of the representative marine bacteria belonging to the *Vibrionaceae* family and is distributed in aquatic environments worldwide (1). Although most *Photobacterium* species are considered nonpathogenic, *P. damselae* (PD) has emerged as a significant pathogen affecting various aquatic organisms such as fish, mollusks, and crustaceans (2). This bacterial species is divided into two subspecies [subsp. *piscicida* (PDP) and subsp. *damselae* (PDD)], of which PDP is known to infect only fish, whereas PDD has been reported to infect marine mammals as well as cold-blooded animals (3, 4). PDD has been reported in fatal or opportunistic infections in several marine mammals, including narrow-ridged finless porpoises (*Neophocaena asiaeorientalis*) (5, 6), common bottlenose dolphins (*Tursiops truncatus*) (7), and Bryde's whales (*Balaenoptera edeni*) (8). PDD is also known to be associated with zoonotic infections in humans, which can cause fatal outcomes, including severe necrotizing fasciitis and septicemia (9–12). Most of those infections are suspected to have been caused by contact with seawater or aquatic animals (13). Moreover, histamine (or scombroid) fish poisoning (HFP) is a type of foodborne intoxication caused by the consumption of seafood that has been spoiled or contaminated by histamine-producing bacteria encoding *histidine decarboxylase*, which plays a crucial role in converting the inherent histidine in seafood to histamine (14, 15). Although no direct evidence of HFP has been reported in free-ranging marine mammals, it is commonly reported in captive pinnipeds and cetaceans due to the ingestion of poorly handled fish feed, causing respiratory congestion (16, 17). Recently, several bacterial genera (or species) have been reported to cause HFP (15, 18, 19), among which PDD is typically considered a strong histamine producer and is one of the most important marine bacterial pathogens responsible for HFP (20). To date, several genomes of pathogenic PDD strains, which have been isolated from various fish or shellfish species, have been sequenced, and their pathogenic features are relatively well understood. However, the microbiological and genomic features of PDD from marine mammals and humans are not yet well understood and require further investigation due to their clinical relevance and potential risks to food safety.

The spotted seal (*Phoca largha*), also referred to as the largha seal, is a pinniped species inhabiting the northern Pacific Ocean and adjacent Arctic seas, and small populations have been recognized in the Yellow Sea and East Sea of Korea (21, 22). Despite being listed as of Least Concern (on the IUCN Red List), the genetically independent population in the Yellow Sea has reduced sharply owing to habitat destruction and marine degradation, putting the population at risk of local extinction (23). Therefore, the Korean government began affording protection status to the spotted seals and banned hunting in the early 1980s, designating the animal as a natural monument in 1982, an endangered species (criterion II) in 2004, and a protected species in 2007 (22). Although several bacteria, fungi, parasites, and viruses have been identified as possible disease-causing agents in captive spotted seals (24–29), research on free-ranging individuals remained limited until recently (30).

Since 2016, we have investigated the emergence of PDD in stranded or diseased marine mammal species present in Korean coastal waters to identify potential pathogens that can colonize and establish fatal infections in these animals for sustainable conservation. The present study describes a case of septicemia in a free-ranging spotted seal (*P. largha*) caused by PDD, with detailed gross histopathological and microbiological features. To the best of our knowledge, this is the first case of PDD infection in *P. largha* and the first report describing the genomic and microbiological characteristics of a PDD isolate originating from a free-ranging pinniped presumed to have caused presumptive systemic infection.

## 2 Materials and methods

### 2.1 Case description

A spotted seal (*P. largha*) was found stranded on a wharf of Gapa Island, Jeju Island, in February 2023. An individual seal (voucher no. CRI012528 deposited at the Cetacean Research Institute (CRI), National Institute of Fisheries Science, Korea) was first seen by residents to be swimming in good health off the Gapa Islet in January 2023, but it was later found dead, and no signs of lethargy or significant external parasites and wounds were observed.

The body was kept with ice in an icebox and transferred to the CRI. Full necropsy and measurement of the whole body were implemented on the two days after stranding. The body was examined from head to tail and from the outside, as per the CRI manual, which was modified from the standard procedure (31), and the internal organs and body cavities were examined *in situ*, then separated from related organs, and measured individually. Representative samples from multiple organs were fixed in 10% neutral formalin and subjected to histological examinations. An additional set of tissues was sampled and individually labeled for ancillary diagnostic studies. Slides for microscopy were prepared by conventional histological techniques, and sections were cut to 5 μm and stained with hematoxylin and eosin. To confirm the potential infection of Japanese encephalitis virus in the seal, the polymerase chain reaction (PCR) was performed on DNA from the frozen organ samples (liver, lungs, lymph nodes, kidney, pancreas, and spleen) using previously published protocols (28), respectively.

### 2.2 Bacterial isolation and identification

During necropsy, sterile swabs were used to collect samples from the kidneys, heart, pericardial fluid, pleural fluid, and blood of the carcass. Bacteria were isolated using a standard dilution plating technique on tryptic soy agar (TSA; BD Difco, MD, USA) and sheep blood agar (BA; Synergy Innovation, Seongnam, Korea), followed by incubation at 37°C for 24 h. Additionally, specialized cultures were performed to screen for the presence of *Vibrio* spp., *Salmonella* spp., *Listeria* spp., and *Brucella* spp., as previously described (32). During the bacterial isolation process, small whitish opaque colonies were predominantly isolated from the examined samples on both TSA and BA and sub-cultured three times for purification. All obtained isolates were stored in tryptic soy broth (TSB; BD Difco, MD, USA) supplemented with 10% glycerol at −80°C until further use.

The isolated bacterial strain was then identified by 16S rRNA sequencing analysis. Bacterial genomic DNA was extracted using a DNeasy Blood & Tissue Kit (Qiagen, Hilden, Germany) according to the manufacturer's instructions. The 16S rRNA gene was amplified using the universal primers 27F and 1492R, and the resulting amplicons were sequenced using the universal primers 785F and 907R at Macrogen Inc. (Seoul, Korea). Biochemical characteristics of the isolate were determined using an API 20E kit (BioMérieux Inc., Marcy-l'Etoile, France) following the manufacturer's instructions to assess the metabolic and enzymatic activities of the bacteria. Furthermore, hemolytic activity was evaluated by inoculating the isolate onto BA, after which the presence or absence of hemolysis was determined. To evaluate the biological characteristics of the isolate obtained from the heart, it and a reference strain, PDD ATCC 51805 (KCTC 12279), were incubated in TSB supplemented with 2.5% NaCl (TSB+). The isolate was incubated at 25 and 37°C, while PDD ATCC 51805 was incubated at 25°C. During incubation, 1mL of bacterial culture was collected at set time points (0, 12, and 24 h) to measure the optical density at an absorbance of 600 nm using a UV/Vis spectrophotometer (K-Lab Co., Daejeon, Korea).

### 2.3. Detection of PD in organs by PCR

PCR was performed to detect the presence of PD in several collected organs (lymph nodes, kidneys, spleen, thyroid, liver, heart, and lungs) and pleural fluids. Total genomic DNA was extracted from the samples using a DNeasy Blood & Tissue Kit (Qiagen, Hilden, Germany) according to the manufacturer's instructions. Then, PCR assays were conducted for the screening of PD-positive samples using the primer set P1 (5′-TAG TGT AGT TAA CAC CTG CAC-3′) and P2 (5′-ACA CTC GAA TCT CTT CAA GT-3′), which were designed to amplify the 16S rRNA gene of PD, following the PCR conditions described (33). The resulting amplicons, ~570 bp in length, were sequenced by Macrogen Inc. (Seoul, Korea) and compared with other PD strains available in the GenBank database using BLASTn searches (www.ncbi.nlm.nih.gov/BLAST).

### 2.4. Antimicrobial susceptibility test

The antimicrobial susceptibility of the isolated PD strain was evaluated according to the testing guidelines and interpretive breakpoints for *Vibrio* spp. described in document M45 from the Clinical and Laboratory Standards Institute (34). Inhibitory zone diameters were determined using antimicrobial susceptibility test discs (Oxoid^®^, Oxoid, NY, USA) containing 19 antimicrobial agents from seven classes: Penicillins and β-Lactam/β-Lactamase inhibitor combinations [Ampicillin (10 μg), Amoxicillin-clavulanic acid (30 μg), Ampicillin-sulbactam (20 μg), Piperacillin (100 μg), and Piperacillin-tazobactam (110 μg)], Cephems [Cefepime (30 μg), Cefotaxime (30 μg), Cefoxitin (30 μg), and Ceftazidime (30 μg)], Carbapenems [Imipenem (10 μg) and Meropenem (10 μg)], Aminoglycosides [Amikacin (30 μg) and Gentamicin (10 μg)], Tetracyclines [Tetracycline (30 μg)], Fluoroquinolones [Ciprofloxacin (5 μg), Levofloxacin (5 μg), and Ofloxacin (5 μg)], Folate pathway inhibitors [Trimethoprim-sultamethoxazole (25 μg)], and Phenicols [Chloramphenicol (30 μg)]. Disk diffusion tests were conducted on Müller–Hinton agar (BD Difco, MD, USA) supplemented with 5% sheep blood (MB cell, Seoul, Korea) at 37°C for 24 h, using *Escherichia coli* ATCC 25922 as a quality control strain.

### 2.5. Genome sequencing analyses

To confirm the exact species (or subspecies) and types of isolated PD strain, the bacterial isolate genome was sequenced using a hybrid approach based on the PacBio Sequel II system (Pacific Biosciences, CA, USA) by constructing a 20-kb SMRTbell^TM^ template library and on the HiSeqXten system (Illumina, CA, USA) by preparing a DNA library using the TruSeq Nano DNA Library Prep Kit (Illumina, CA, USA). The Microbial Genome Assembly application (http://www.pacb.com/products-and-services/pacbio-systems/sequel/sequel-software/) was used to assemble PacBio long reads (1,088,423,107 bp; 110,677 reads) and Illumina paired-end reads (2,574,112,928 bp; 17,055,440 reads) for error correction using Pilon v1.21 (35). Genome annotation was performed using the National Center for Biotechnology Information Prokaryotic Genome Annotation Pipeline (https://www.ncbi.nlm.nih.gov/genome/annotation_prok/). To determine the exact species (or subspecies) of the isolated PD strains, *gyrB* and *toxR* genes were searched from the genome and aligned with those sequences from representative type strains of *Photobacterium* species in the GenBank database using ClustalX (v2.1) (36) and BioEdit (v7.1.0.3) (37). The datasets were then phylogenetically analyzed using MEGAX (v10.0) (38). Phylogenetic trees were constructed using the maximum likelihood (ML) method, and the reliability of the trees was assessed using 1,000 bootstrap replicates. Additionally, whole genome-based phylogenetic analyses were performed using the Type (Strain) Genome Server (TYGS) (https://tygs.dsmz.de/). To assess the genomic relatedness of the isolated PD strains to other *Photobacterium* species, average nucleotide identity (ANI) was analyzed using OrthoANI (https://www.ezbiocloud.net/tools/orthoani) against several other type strains of the genus, excluding PDP, available in the GenBank database. Genomic analyses were conducted using the Pathosystems Resource Integration Center (PATRIC) to visualize and organize genome functions (39). For the genomic characterization of the plasmid in the isolated PD strain, we first compared the plasmid by direct sequence comparison using BLASTn search with other previously reported PD plasmids available in the GenBank database. Then, a detailed comparative genomic analysis of the plasmid of the isolated PD strain was performed using BRIG (BLAST Ring Image Generator) (40) to compare and visualize with highly similar plasmids previously reported in PD strains.

The major virulence factors specifically associated with PD strains (41) and gene clusters associated with histamine production (6), including histidine/histamine antiporters, histidine decarboxylase, and histidyl-tRNA synthetase, were identified by manual comparisons with those available in the GenBank database. Putative virulence-associated genes were screened by searching against the Virulence Factor Database (http://www.mgc.ac.cn/VFs/). Moreover, the presence of genetic determinants related to antimicrobial resistance was screened using the Comprehensive Antibiotic Resistance Database (https://card.mcmaster.ca/). PHASTEST (http://phastest.ca/) was used to detect prophages in the genome.

### 2.6. Determination of histamine-producing ability of PDD isolate

To evaluate the histamine-producing ability of the identified PDD isolate, two PDD strains, including the isolated PDD strain and the reference strain PDD ATCC 51805, were prepared as follows: both the PDD strains were cultured on TSA supplemented with 2.5% NaCl, with the PDD isolate incubated at both 25 and 37°C, and PDD ATCC 51805 at 25°C. Fresh cultures of each strain were inoculated (1%, v/v) into 40 mL aliquots of TSB+ supplemented with 1% L-histidine. The inoculated broths were then incubated in a shaking incubator (Vision Scientific, Daejeon, Korea) at the respective temperatures for 24 h. A negative control group was prepared by inoculating TSB+ with sterile distilled water without a bacterial culture. At predetermined time points (0, 12, and 24 h) during incubation, 1 mL of each sample was collected from each culture and mixed with 9 ml of 0.1 M EDTA (pH 8.0) solution. The mixtures were then centrifuged at 12,000 rpm for 5 min to obtain supernatants. The collected supernatants were filtered using a 0.22 μm syringe filter (JetBiofil^®^, Guangzhou, China), and the histamine concentration in each sample was determined using a commercial histamine test kit (Kikkoman Biochemifa, Tokyo, Japan) according to the manufacturer instructions. All experiments were performed in triplicates.

### 2.7. Bacterial cytotoxicity assay

The cytotoxic effects of the PDD isolate on mammalian cells were evaluated using a Quanti-Max WST-8 cell viability assay kit (Biomax Ltd., Seoul, Korea) according to the manufacturer's instructions with some modifications. The human fibrosarcoma epithelial cell line HT-1080 (KCLB 10121) was obtained and maintained in RPMI 1640 medium (Welgene Inc., Gyeongsan, Korea) supplemented with 300 mg/L L-glutamate, 25 mM HEPES, 2,000 mg/L sodium bicarbonate, 10% heat-inactivated fetal bovine serum (FBS; Median Life Science, Houston, TX, USA), and 1% penicillin-streptomycin (Welgene Inc., Gyeongsan, Korea). The cells were cultured at 37°C in a humidified atmosphere containing 5% CO_2_. For the cytotoxicity assays, HT-1080 cells were seeded into 96-well flat-bottomed plates (SPL, Seoul, Korea) at a density of 5 × 103 cells/well and incubated for 24 h at 37°C under 5% CO_2_ to allow cell attachment. The cells were then exposed to 10 μL of the pre-cultured PDD isolate at different concentrations (10, 10^2^, 10^3^, 10^4^, 10^5^, and 10^6^ CFU/mL) and incubated for an additional 24 h at 37°C under 5% CO_2_. Control groups were inoculated with phosphate-buffered saline (Welgene Inc., Gyeongsan, Korea) without bacterial culture. After incubation, the morphological changes in the cells were observed and captured using a Eclipse Ti-S inverted microscope (Nikon, Tokyo, Japan). The cell viability was assessed by adding 10 μL of WST-8 reagent to each well and incubating the plates for 30 min at 37°C. The optical absorbance was measured at 450 nm using a microplate reader (Molecular Devices, San Jose, CA, USA). All experiments were performed in triplicates.

### 2.8. Nucleotide sequences deposition

The PDD strain GCUPdd isolated in this study was deposited with the Korean Culture Center of Microorganisms (KCCM) under deposition number KCCM 90590. The complete genome sequence of the PDD strain GCUPdd has been deposited in the GenBank database under accession numbers CP165601 (chromosome 1), CP165602 (chromosome 2), and CP165603 (plasmid).

## 3 Results

### 3.1. Gross and histological findings

The stranded seal individual was identified based on the morphological characteristics and was finally confirmed to be a spotted seal (*P*. *largha*). The seal weighed 46.8 kg, was a 138.4 cm long female, and was assumed to be an immature one based on the body length and developmental stage of the reproductive organs. Although several scarring and healed wounds were observed on the body, no skeletal fractures or contusions that may have contributed to death were observed. Subcutaneous fat thickness (abdomen) was 37 mm, indicating a generally healthy body condition.

**Figure 1:**
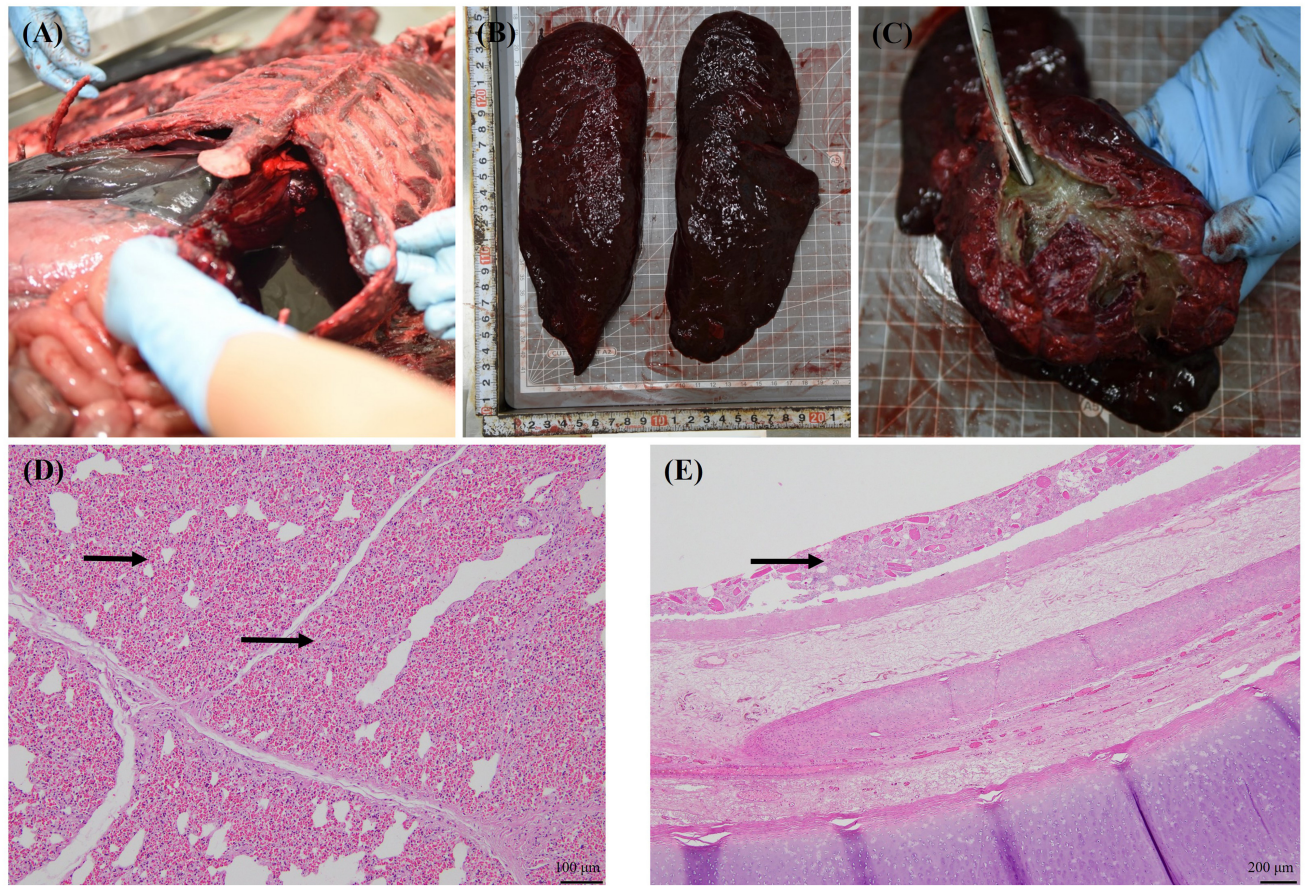
Gross and histological findings in the stranded spotted seal (voucher no. CRI012528). Bloody fluid in the thoracic cavity **(A)**; Lungs with parenchymal congestion and underinflation **(B)**; The mucosa of trachea and bronchi were covered with yellow to dark green muddy substances and no bloody foam in the airway **(C)**; Severe diffuse pulmonary congestion (arrow) and alveolar cavity reduction secondary to the congestion **(D)**; Foreign material present within the tracheal mucosa (arrow) **(E)**.

Major gross findings in the seal included marked hemorrhagic pleural effusion in the thoracic cavity, congestion of the small and large intestines and lungs, and no foam in the trachea, with widespread mud-like, yellowish to dark green substances covering the tracheal mucosa (Figures 1A–C). Both lungs exhibited parenchymal congestion, accompanied by suggestive features of underinflation, and similar muddy substances covered the bronchioles. Two heartworms were found, one in the right ventricle and the other at the entry of the pulmonary artery. The stomach and intestines were filled with undigested fish, mild congestion of the mucosa was observed in the lower part of the large intestine, and no air embolism was identified in the mesenteric lymph nodes or mesenteric blood vessels. No notable findings were observed in the reproductive organs, liver, kidneys, or urinary system.

Histological findings were as follows: the lungs exhibited a severe edematous condition that caused alveolar contraction (Figure 1D). Foreign materials were found from the trachea to the bronchioles (Figure 1E); however, there was no relationship with inflammation, and these were considered postmortem. No other special signs were observed in other organs.

### 3.2. PDD caused presumptive systemic infection in *P. largha*

**Figure 2:**
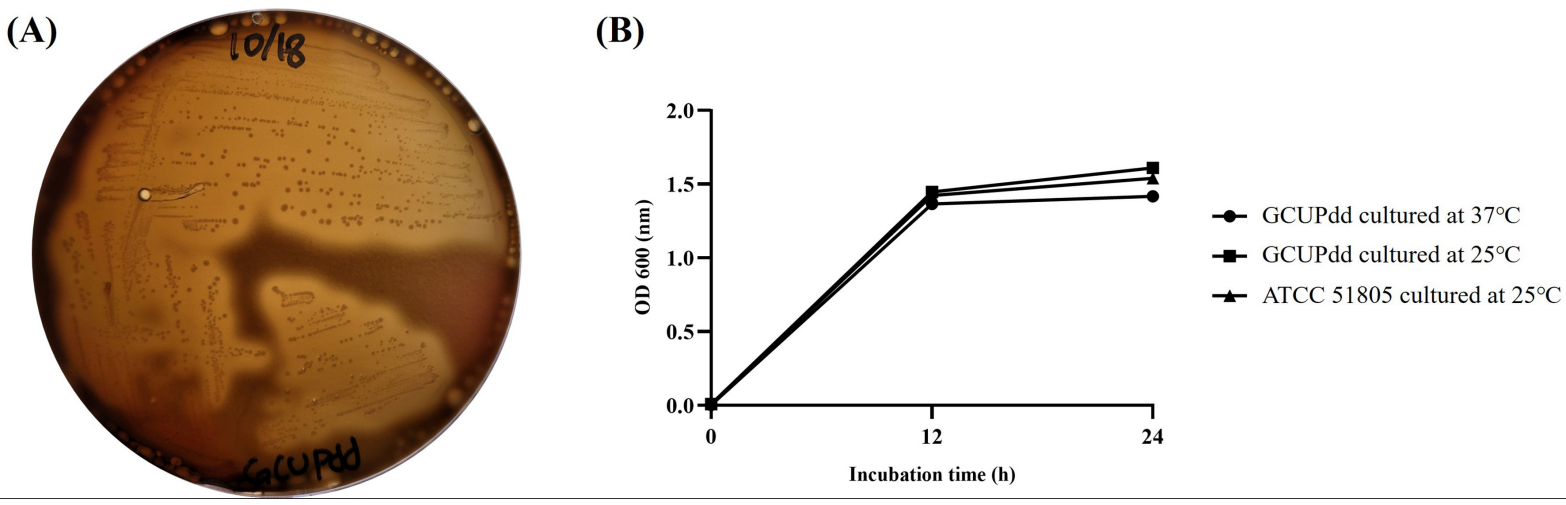
Biological characteristics of PDD strain GCUPdd. **(A)** Hemolysis test of strain GCUPdd on sheep blood agar, showing strong β-hemolysis after 24 h of incubation at 37°C. **(B)** Growth curves of PDD strain GCUPdd and reference strain ATCC 51805 cultivated in TSB supplemented with 2.5% NaCl. The growth curve experiments were performed in triplicate, and error bars represent standard deviation.

Small whitish opaque colonies were commonly isolated from the kidneys, heart, and pericardial fluid of the seal carcass during bacterial isolation. These isolates were non-luminescent, gram-negative, rod-shaped bacteria that were oxidase- and catalase-positive. They resulted in strong β-hemolysis on 5% BA after 24 h of incubation at 37°C (Figure 2A). Based on 16S rRNA sequencing analysis, the isolate showed > 99% nucleotide sequence similarity with other PD strains available in the GenBank database. PD was the only bacterial species consistently isolated in significant numbers from the spotted seal-sampled organs during routine bacterial culture. Additionally, no growth of other potentially pathogenic bacteria including *Vibrio* spp., *Salmonella* spp., *Listeria* spp., or *Brucella* spp. was observed on selective or enriched media from the collected samples of internal organs and body fluids. Moreover, PCR tests for *Brucella* spp. and Japanese encephalitis viruses were negative. Since all of the confirmed PD isolates exhibited growth ability at 37°C (Figure 2B), which is an inhibitory temperature for PDP, and hemolysis on BA, the PD isolates from the seal in this study were finally classified as PDD. The representative PDD strain, designated as strain GCUPdd, which was isolated from the heart of the spotted seal, was selected for further analysis. Biochemical tests revealed that strain GCUPdd was urease-positive (Supplementary Table S1).

A PCR assay using the primer sets P1/P2, targeting the highly conserved region of 16S rRNA of PD, successfully amplified c.a. 570 bp nucleotide fragments from PDD strain GCUPdd. This isolate was then used as a positive control for PCR analysis. PCR-based screening detected the presence of PDD in several organs (heart, kidney, lung, lymph nodes, spleen, and thyroid) and pleural fluid, with amplicons identical to those of strain GCUPdd. This finding indicated that the bacteria might have caused a systemic infection in the seal (Supplementary Figure S1). The antimicrobial susceptibility profile of PDD strain GCUPdd was evaluated based on the CLSI guidelines. GCUPdd was resistant to ampicillin, intermediate to meropenem, and susceptible to other tested antibiotics. The antimicrobial resistance profiles of GCUPdd are summarized in Table 1.

**Table 1:** Antimicrobial susceptibility of PDD strain GCUPdd using the disk diffusion test.

**Strain**	**Antimicrobial agents [disk content (**μ**g)]**																		
**PDD GCUPdd**	**PL**					**Cep**				**Carb**		**Am**		**Tet**			**Fq**	**Fpi**	**P**
	AMP (10)	AMC (30)	SAM (20)	PRL (100)	TZP (110)	FEP (30)	CTX (30)	FOX (30)	CAZ (30)	IPM (10)	MEM (10)	AK (30)	CN (10)	TE (30)	CIP (5)	LEV (5)	OFX (5)	SXT (25)	C (30)
	13	25	28	30	36	30	36	30	35	34	20	24	20	36	34	36	32	16	34

A category of antibiotic susceptibility is dark gray, resistant; light gray, intermediate; white, susceptible. PL, Penicillins and β-Lactam/β-Lactamase inhibitor combinations; Cep, Cephems; Carb, Carbapenems; Am, Aminoglycosides; Tet, Tetracyclines; Fq, Fluoroquinolones; Fpi, Folate pathway inhibitors; P, Phenicols; AMP, Ampicillin; AMC, Amoxycillin-clavulanic acid; SAM, Ampicillin-sulbactam; PRL, Piperacillin; TZP, Piperacillin-tazobactam; FEP, Cefepime; CTX, Cefotaxime; FOX, Cefoxitin; CAZ, Ceftazidime; IPM, Imipenem; MEM, Meropenem; AK, Amikacin; CN, Gentamicin; TE, Tetracycline; CIP, Ciprofloxacin; LEV, Levofloxacin; OFX, Ofloxacin; STX, Trimethoprim-sultamethoxazole; C, Chloramphenicol.

### 3.3. Genome of PDD strain GCUPdd revealed pathogenic features

**Figure 3:**
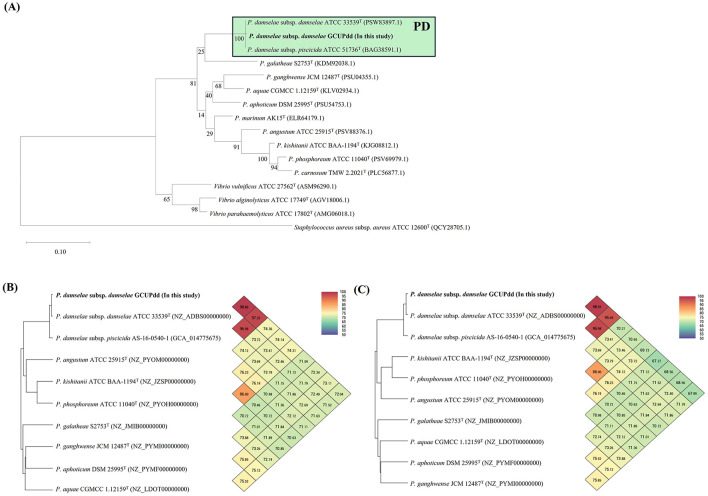
The single gene-based **(A)** and genome-based **(B, C)** identifications of the PDD strain GCUPdd using those from representative reference strains of *Photobacterium* spp., which are currently available in the GenBank database. A maximum-likelihood phylogenetic tree was reconstructed based on the amino acid sequences of the *gyrB* gene **(A)**. The scale bar represents 0.10 amino acid substitutions per site, and *Staphylococcus aureus* ATCC 12600^T^ was used as an outgroup. Orthologous average nucleotide identity (orthoANI) heatmaps of PDD strain GCUPdd **(B, C)**. The orthoANI heatmaps are shown for chromosome I **(B)** and chromosome II **(C)**, and each pairwise comparison values between strains are summarized at the points where their respective diagonal lines intersect.

**Figure 4:**
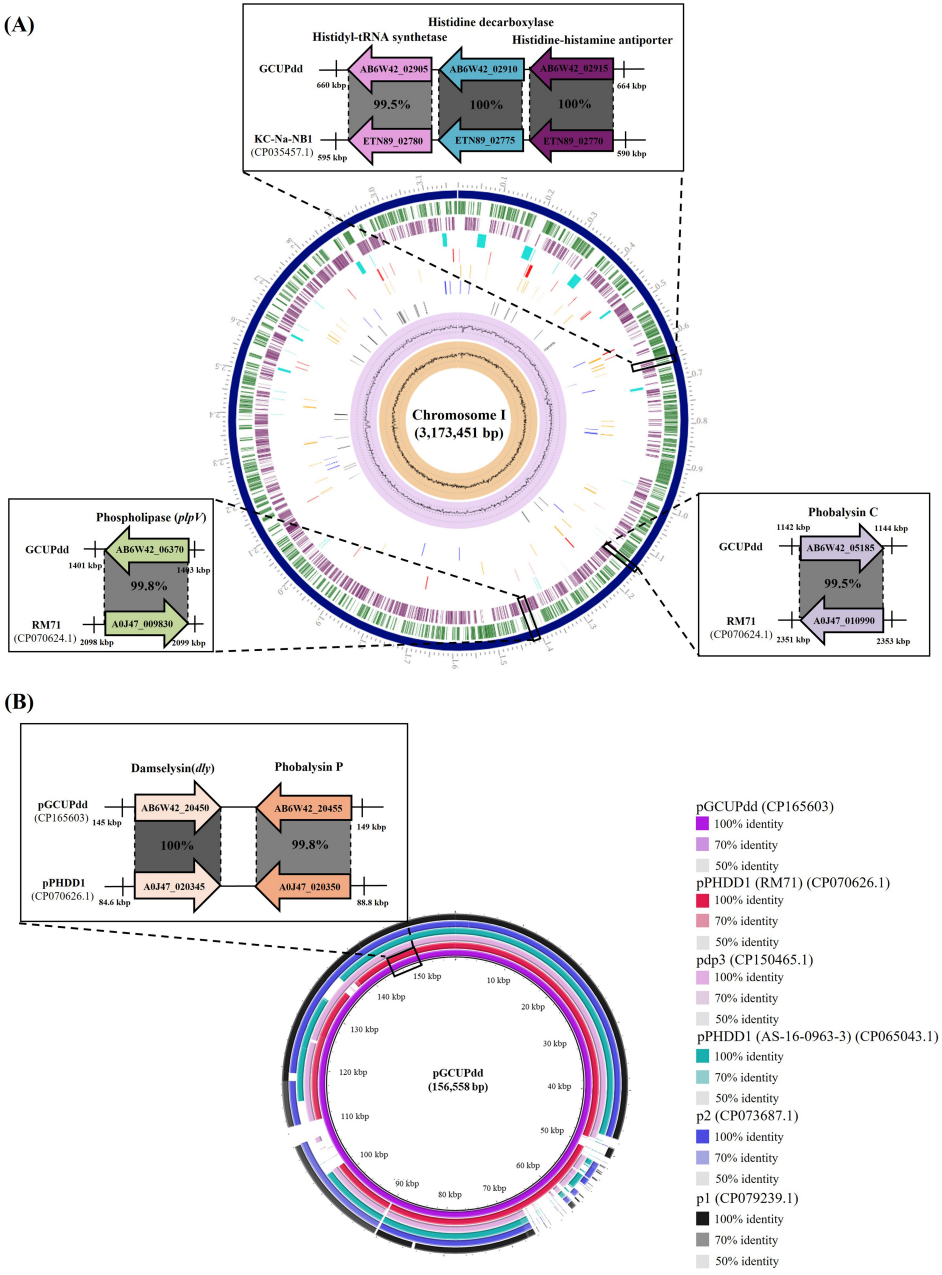
Comparison of the virulence-related genes (or gene clusters) in chromosome **(A)** and plasmid **(B)** of the PDD strain GCUPdd with those of the reference PDD strains. The chromosome I **(A)** of the PDD isolate contained phospholipase, phobalysin C, and a histamine production-related gene cluster, which are almost identical to those previously reported in the PDD strains, respectively. The plasmid pGCUPdd **(B)** contained damselysin and phobalysin P, which are almost identical to those previously reported in the plasmid pPHDD1, respectively. The figure highlights these genomic regions and provides enlarged views for detailed comparisons of the relevant regions with reference PDD strains and pPHDD1-type plasmids. Coding sequences are represented by arrows indicating direction and annotations. The amino acid identity values based on BLASTp analysis are provided for each gene, showing the identity between the strain GCUPdd and the reference PDD strains.

The complete genome of PDD strain GCUPdd comprised two chromosomes (chromosome I: 3,173,451 bp with 41.5% G+C content; chromosome II: 1,222,862 bp with 39.1% G+C content) and one plasmid (pGCUPdd: 156,558 bp with 37.8% G+C content) (Supplementary Figure S2). The annotated genome contained 4,095 genes, 3,778 protein-coding sequences, 62 rRNAs (5S, 16S, and 23S), and 209 tRNAs. Phylogenetic analyses based on *gyrB* and *toxR* were performed to determine the exact species and subspecies of strain GCUPdd. The *gyrB* gene (2,415 bp, locus_tag: AB6W42_14605)-based phylogeny showed that strain GCUPdd clustered together with the type strains PDD and PDP and was distinctly separated from other *Photobacterium* species (Figure 3A). The *toxR* gene (903 bp, locus_tag: AB6W42_04560) phylogeny further confirmed that strain GCUPdd belonged to PDD, clearly distinguishing it from the PDP strains (Supplementary Figure S3A). Whole genome-based phylogenetic trees also supported the classification of the GCUPdd strain as a member of the PDD grouping (Supplementary Figures S3B, C). OrthoANI was used to assess genome similarity between the PDD strain GCUPdd and representative *Photobacterium* species. Strain GCUPdd showed >96% ANI values against representative PD genomes, with the highest ANI value (98.9%) observed against the PDD-type strain (Figures 3B, C). Although the absence of a PDP-type strain genome limited the precision of our analysis, strain GCUPdd exhibited distinct differences from the type strains of other *Photobacterium* species, supporting its classification as a PDD based on phenotypic and genetic characteristics. The primary PDD virulence factors phobalysin C (locus_tag: AB6W42_05185) and phospholipase (*plpV*) (locus_tag: AB6W42_06370) were encoded on chromosome I of strain GCUPdd, showing 99.8 and 99.5% amino acid identity, respectively, with those found in the hemolytic PDD strain RM-71 which was isolated from diseased turbot (*Scophthalmus maximus*) (41). Moreover, a gene cluster on chromosome I containing a histidyl-tRNA synthetase (locus_tag: AB6W42_02905), a histidine decarboxylase (locus_tag: AB6W42_02910), and a histidine-histamine antiporter (locus_tag: AB6W42_02915) shared >99% amino acid identity with functionally characterized genes in PDD KC-Na-NB1 (CP035457.1) (Figure 4A).

The direct sequence comparison using BLAST search revealed that the plasmid pGCUPdd (156,558 bp) was most similar to the plasmid pPHDD1 (CP070626.1), a 153-kb conjugative virulence-associated plasmid originally identified in the PDD strain RM-71 (41), with 99.1% nucleotide similarity (89.0% query coverage). The plasmid pGCUPdd also showed >96.5% nucleotide similarity to other pPHDD1-like plasmids previously found in PDD (p1 (CP079239.1), p2(CP073687.1), and pPHDD1 (CP065043.1) from strain AS-16-0963-3) and PDP (pdp3 (CP150465.1). Damselysin (*dly*) (locus_tag: AB6W42_20450) and phobalysin P (locus_tag: AB6W42_20455), identified as the main PDD pathogenic factors in the pPHDD1 and pPHDD1-like plasmids, were also detected on the plasmid pGCUPdd, showing 100 and 99.8% amino acid identity (Figure 4B), respectively. However, the plasmid pGCUPdd was 3,129 bp longer than the plasmid pPHDD1, and its additional sequence region contained several genes absent in the plasmid pPHDD1, including a type VI secretion system valine-glycine repeat (Vgr) family protein (locus_tag: AB6W42_20265), a hemolysin coregulated protein (Hcp) family type VI secretion system effector (locus_tag: AB6W42_20270), and a GIY-YIG nuclease family protein (locus_tag: AB6W42_20415). In contrast, a PAAR domain-containing protein (locus_tag: A0J47_020130), which has been found in the plasmid pPHDD1, was not predicted in the plasmid pGCUPdd.

Screening for the additional bacterial virulence-associated genes using the virulence factor database identified various factors in the GCUPdd genome, including those related to pili, flagella, secretion systems, thermolabile hemolysin (*tlh*), and immune evasion mechanisms (Supplementary Table S2). Additionally, seven antimicrobial-resistance-related genes, including antibiotic efflux pumps, were detected on the chromosome (Supplementary Table S3). No prophage regions were identified using PHASTEST analysis. These findings strongly suggest that the strain GCUPdd may have pathogenic potential in aquatic animals, including marine mammals and humans.

### 3.4. PDD strain GCUPdd possessed strong histamine-producing potential

**Figure 5:**
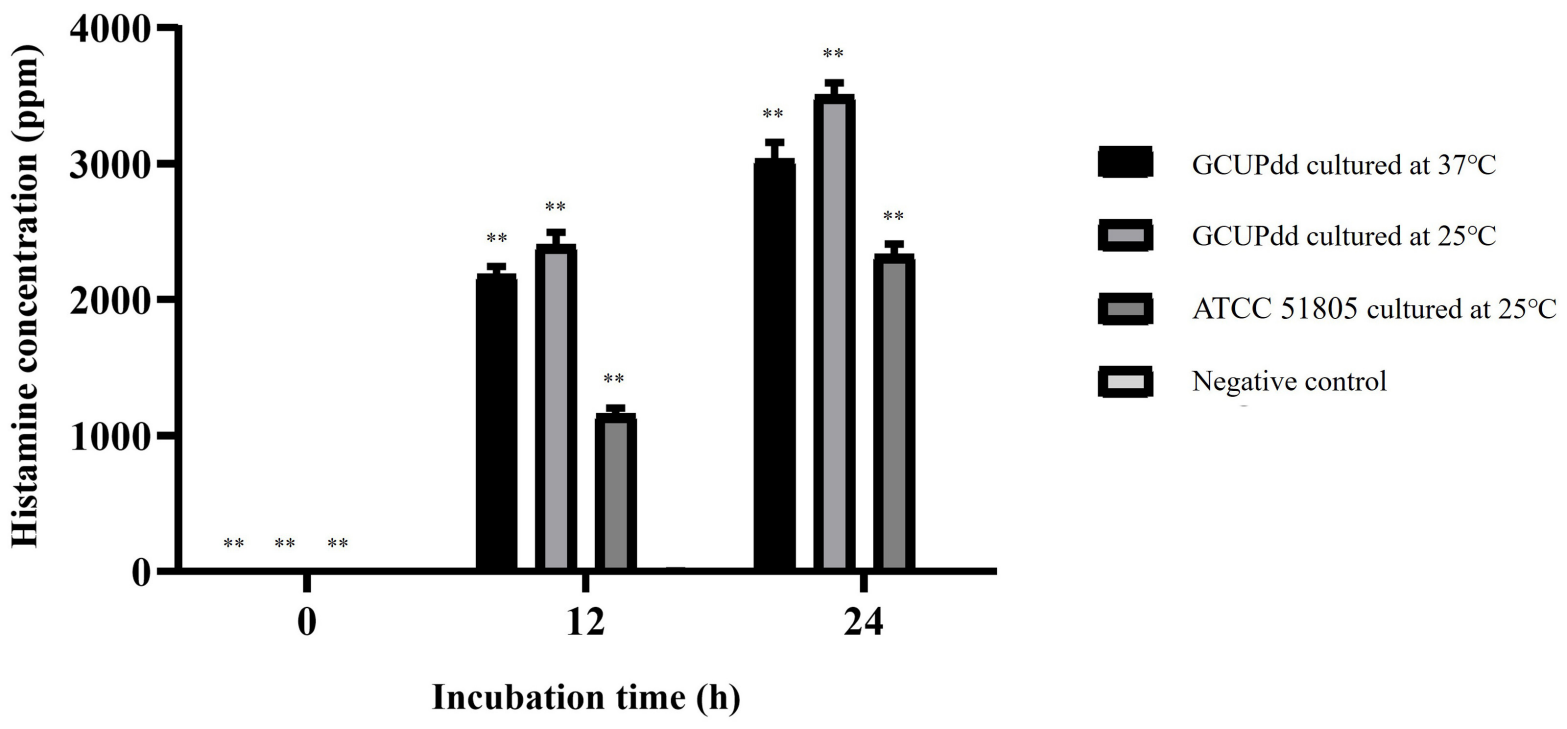
Histamine production by PDD strain GCUPdd and reference strain PDD ATCC. Histamine concentrations were measured at 0, 12, and 24 h of incubation in TSB+ with 1% L-histidine. The strain GCUPdd was incubated at both 25 and 37°C,while strain ATCC 51805 was incubated at 25°C. All experiments were performed in triplicate and error bars represent standard deviation (**p < 0.01).

The histamine-producing ability of the PDD strain GCUPdd was evaluated and compared to that of the reference strain PDD ATCC 51805. The results showed that strain GCUPdd exhibited higher histamine production than strain ATCC 51805 at all-time points (Figure 5). After 12 h of incubation, strain GCUPdd produced ~2,100–2,400 ppm of histamine, whereas strain ATCC 51805 produced ~1,100 ppm, indicating a significant increase in the apparent histamine concentration of the strain GCUPdd (*p* < 0.01). After 24 h of incubation, the histamine concentrations reached ~3,000–3,500 ppm for strain GCUPdd and 2,300 ppm for strain ATCC 51805, further demonstrating the significantly higher histamine-producing ability of strain GCUPdd (*p* < 0.01). Interestingly, the strain GCUPdd cultured at 25°C consistently produced ~15% more histamine than the strain GCUPdd cultured at 37°C, both after 12 and 24 h. This finding suggests that the PDD strain GCUPdd could have strong potential for histamine production and its production could be affected by the incubation temperature, with a lower temperature (25°C) promoting greater histamine production compared to a higher temperature (37°C).

### 3.5. PDD strain GCUPdd possessed potential cytotoxicity

**Figure 6:**
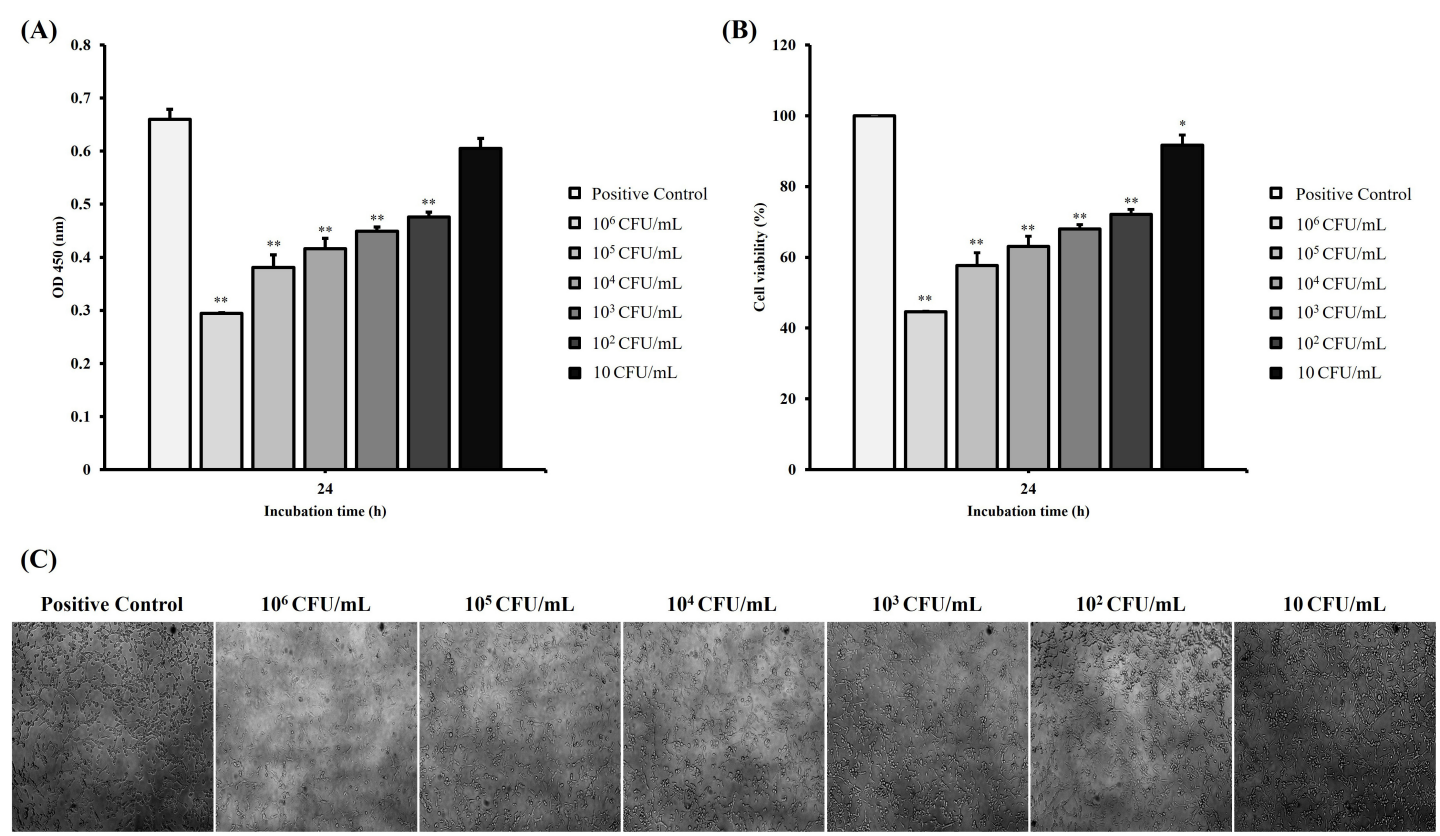
Cytotoxic effects of PDD strain GCUPdd on human fibrosarcoma epithelial cell line HT-1080. Cell viability of HT-1080 cells exposed to different concentrations of strain GCUPdd (10, 10^2^, 10^3^, 10^4^, 10^5^, and 10^6^ CFU/mL) at 37°C for 24h, presented as OD_450_ values **(A)** and cell viability (%) **(B)**, respectively. Cells treated with PBS only were used as a negative control. The experiments were performed in triplicate, and error bars represent standard deviation. **(C)** Morphological changes of HT-1080 cells exposed to strain GCUPdd at different concentrations for 24 h, as observed using a Nikon Eclipse Ti-S inverted microscope (*p < 0.05; **p < 0.01).

To assess the virulence potential of PDD strain GCUPdd, its cytotoxicity was investigated by measuring cell viability of the human fibrosarcoma epithelial cell line HT-1080 in response to bacterial exposure. The viability of HT-1080 cells was significantly different when treated with different concentrations of strain GCUPdd compared to that of the control (Figure 6). The exposure of HT-1080 cells to strain GCUPdd resulted in a concentration-dependent increase in cytotoxicity, which was associated with a significant decrease in cell viability. The viability of HT-1080 cells was significantly reduced to 56%, 43%, 37%, 32%, and 28% at bacterial concentrations of 10^6^, 10^5^, 10^4^, 10^3^, and 10^2^ CFU/mL, respectively (*p* < 0.01). HT-1080 cell viability was reduced by > 50% within 24 h of exposure to 10^6^ CFU/mL of strain GCUPdd. As expected, HT-1080 cells exhibited morphological changes when exposed to strain GCUPdd compared with the control (Figure 6C). Cell density decreased and the size of most cells in the bacterial exposure group was reduced compared to that in the control. Microscopic images of HT-1080 cells treated with strain GCUPdd clearly demonstrated the cytotoxic effects of the bacteria, with a notable reduction in cell number and altered cell morphology.

## 4 Discussion

This study reports the first case of presumptive systemic infection caused by PDD in a free-ranging spotted seal (*P*. *largha*) stranded in Korea. The microbiological, genomic, and pathogenic characterization of the PDD isolate (strain GCUPdd) provided new insights into the potential of this bacterial pathogen in marine mammalian diseases.

The gross pathological findings in the spotted seal, including hemorrhagic pleural effusion and congestion of the intestines and lungs, were consistent with acute septicemia. The isolation of PDD, along with the absence of other significant bacterial or viral pathogens from multiple organs (kidney, heart, and pericardial fluid) and PCR detection in the thyroid, heart, liver, and blood strongly support PDD as the etiological agent of the systemic infection in this case. These findings have been predominantly limited to cetaceans, as previously reported PDD-causing fatal septicemia in other marine mammals such as narrow-ridged finless porpoises (*N. asiaeorientalis*), common bottlenose dolphins (*T. truncatus*), and Bryde's whales (*B. edeni*) (5–8). The confirmation of PDD infection in spotted seals expands the known host range of this pathogen among pinnipeds, highlighting its adaptability to diverse marine mammal hosts and raising concerns regarding its potential impact on pinniped populations. To our knowledge, this is the first report of PDD infection in a spotted seal.

The isolated PDD strain GCUPdd exhibited hemolytic activity and growth at 37°C, key phenotypic characteristics distinguishing PDD from the fish-specific PDP (1). Genome sequencing confirmed the identity of the GCUPdd subspecies. The *gyrB* gene, which is widely used for phylogenetic analyses within the *Vibrionaceae* family, clearly clustered strain GCUPdd with PDD- and PDP-type strains, distinct from other *Photobacterium* species (1). The *toxR* gene is considered an important subspecies-differentiating marker in PD due to the significant sequence divergence between PDD and PDP (7, 42), further confirming that strain GCUPdd is PDD. Whole genome ANI analyses revealed values exceeding the 95–96% cut-off threshold for species demarcation (43–45), supporting the classification of strain GCUPdd within the PDD. Importantly, chromosome I of the strain GCUPdd encodes major PDD virulence factors, including phobalysin C and *plpV*, which potentially contribute to the high pathogenicity of the strain. Moreover, the plasmid pGCUPdd in the PDD isolate was very similar (>99% nucleotide similarity) to the plasmid pPHDD1, originally identified in the hemolytic and pathogenic PDD strain RM-71, and also harbored the damselysin and phobalysin P, which have been shown to act synergistically in disrupting host cell membranes and inducing tissue damage (41). These results indicate the conservation of core virulence factors in the strain GCUPdd, thus suggesting its potential to cause systemic dissemination during infection. Different from the type VI secretion system (T6SS)-related genes in the plasmid pPHDD1, the plasmid pGCUPdd encoded additional components of the T6SS, including Vgr family protein and Hcp family effector, which have been reported to be essential for the assembly of a functional T6SS and the delivery of associated effectors (46, 47). Although the function of T6SS has not yet been fully characterized in PDD, the T6SS in related *Vibrionaceae* species has been reported to play a key role in interbacterial competition by delivering toxic effectors to competing bacteria (48). Therefore, the presence of both core virulence factors and T6SS-related genes in the plasmid GCUPdd suggests that this PDD strain might be capable of damaging host cells and competing with other bacteria, which may contribute to its pathogenicity in marine environments. Moreover, GCUPdd exhibited significant cytotoxicity against human cells in a concentration-dependent manner, resulting in marked cell rounding and death. These results strongly supported the pathogenic potential of this strain and the ability of PDD to cause severe necrotizing infections in humans following wound exposure (9, 11).

Interestingly, the GCUPdd genome also harbors a gene cluster with high homology to the histidine decarboxylase pathway, which is involved in histamine production. PD is recognized as a major bacterial histamine producer and a causative agent of scombroid poisoning in seafood (20). Histamine-producing-functional assays confirmed the ability of strain GCUPdd to produce high levels of histamine, exceeding that of a reference strain, particularly at a lower incubation temperature (25°C), compared to other known potent histamine-producing bacteria, such as *Morganella morganii* and *Klebsiella pneumoniae* (49, 50). While there is no direct evidence of histamine poisoning in free-ranging marine mammals, this may be due to a failure to recognize the problem rather than resistance to histamine toxicity. Suspected clinical cases have been reported in captive bottlenose dolphins, ringed seals, California sea lions, and killer whales (*Orcinus orca*), with symptoms ranging from recurring “sore throat” episodes to respiratory congestion and performance refusal (16). These cases were attributed to the ingestion of spoiled fish because the symptoms were consistent with the inflammatory responses of high histamine levels (15). The potential contribution of bacterial histamine production to the pathogenesis of septicemia in marine mammals, as well as its role in histamine-related foodborne illnesses, such as scombroid fish poisoning (51), requires further investigation. The ability of strain GCUPdd to produce high histamine levels at lower temperatures is particularly relevant because improper storage of fish feed can lead to histamine accumulation.

To date, the spotted seal *P*. *largha* has been classified as being at risk of local extinction in the Yellow Sea due to human impacts (23). However, the potential pathogens that can affect and threaten animal health remain poorly understood. Some parasitic or viral pathogens, including *Dirofilaria immitis* and Japanese encephalitis virus, have been reported in spotted seals in Korea (28). In marine mammals, PDD has been isolated from several stranded dolphins and whales with or without associated lesions, and the pathogenic role of PDD in the animals remains unclear (3, 8, 52–55). Similar to the cetaceans, we cannot easily determine the specific position of PDD, whether it is commensal or pathogenic to marine pinnipeds, particularly in *P*. *largha*, owing to the lack of reports describing its isolation. Nevertheless, the isolation of PDD with cytotoxic and histamine production capabilities from free-ranging *P*. *largha* in this study indicates the potential clinical relevance of PDD in pinnipeds and also highlights the importance of monitoring pathogens in marine mammals, especially in spotted seals. Moreover, as PDD exhibits strong pathogenic potential by causing histamine poisoning, severe skin necrosis, and blood infections in humans (13), the zoonotic potential of PDD from free-ranging marine mammals needs to be further investigated to reduce its impact on veterinary and public health. In fact, strain GCUPdd exhibited resistance or reduced susceptibility to certain β-lactam antibiotics, which have been used to treat human PDD infection (13). Therefore, continued surveillance of PDD in marine environments is essential to conserve free-ranging marine mammals and prevent zoonotic infections and histamine poisoning in fish.

## Data Availability

The data presented in this study are deposited in the following repositories: Figshare, https://doi.org/10.6084/m9.figshare.29832863.v1, GenBankdatabase (https://www.ncbi.nlm.nih.gov/), CP165601 (chromosome 1), CP165602 (chromosome 2), and CP165603 (plasmid).
