# Supplementary material for: Characterization of Photobacterium damselae subsp. damselae isolated from a spotted seal (Phoca largha) (Pinnipedia: Phocidae) stranded in Korea
Source: Front Vet Sci. 2025 Aug 7;12:1574705. doi: 10.3389/fvets.2025.1574705 (PMC12369411; doi:10.3389/fvets.2025.1574705)
Supplement: Supplementary file 1 [file Table_1.DOCX]

Supplementary Material

#
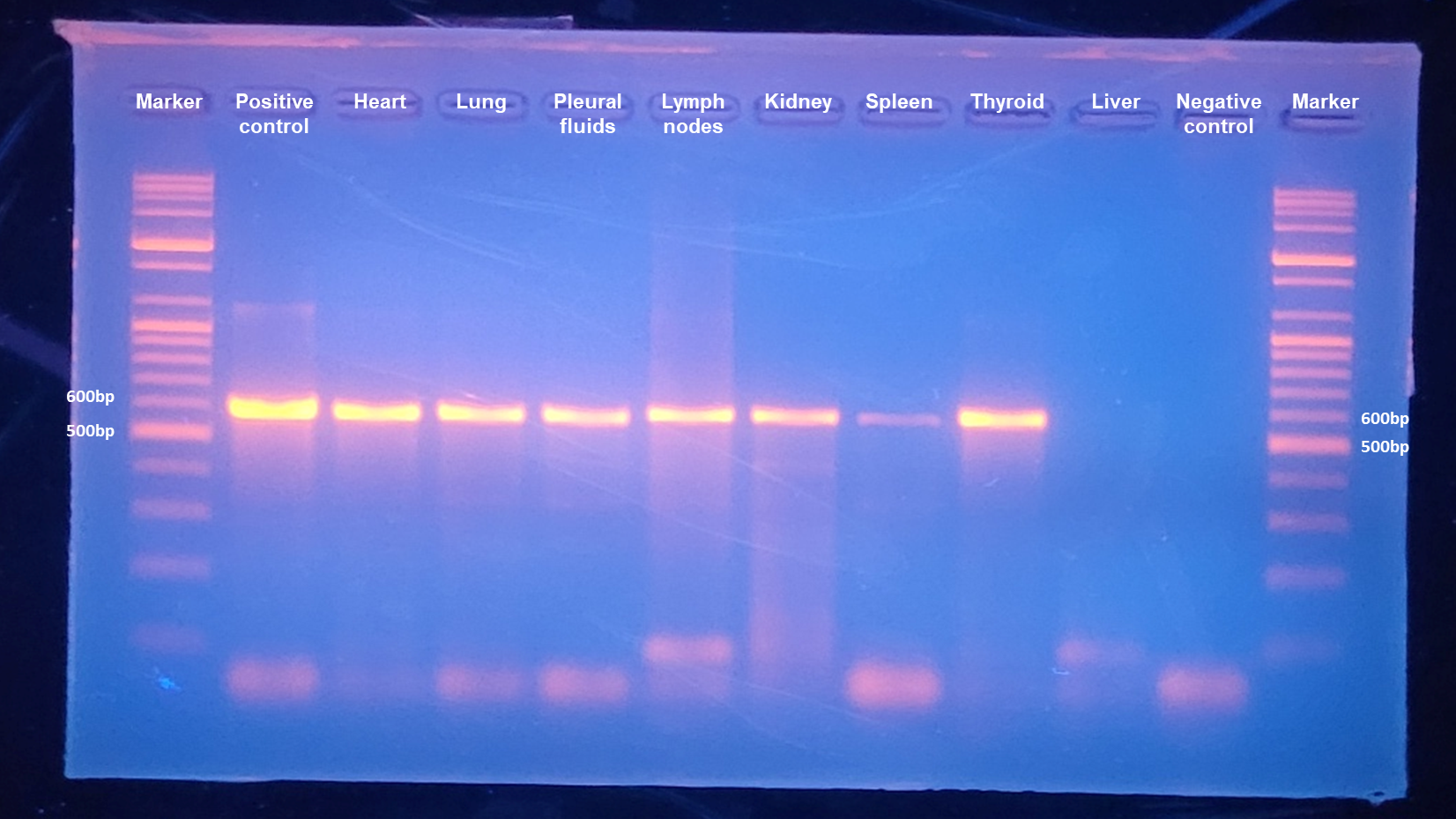


# Figure S1: PCR-based screening on the presence of PDD in the seal CRI012528. The c.a. 570 bp PCR amplicons of PDD were detected in the collected organs (heart, kidney, lung, lymph nodes, spleen, and thyroid) and the pleural fluids, with amplicons identical to those of the PDD strain GCUPdd (positive control)

#
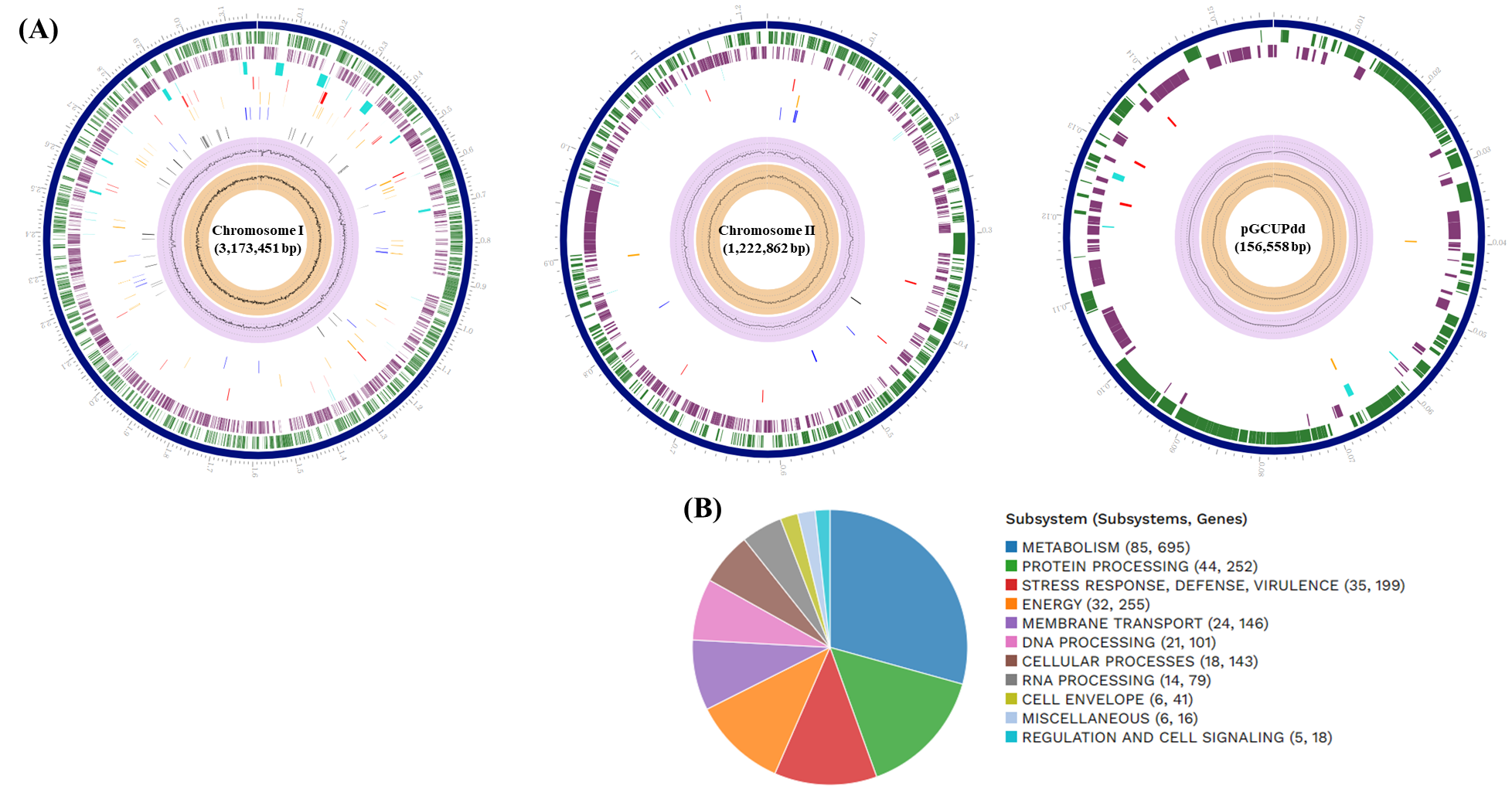


# Figure S2: Genome of PDD strain GCUPdd, annotated and represented as a circular graphical map (A), and its subsystems (B) using the Pathosystems Resource Integration Center (PATRIC) server;

#
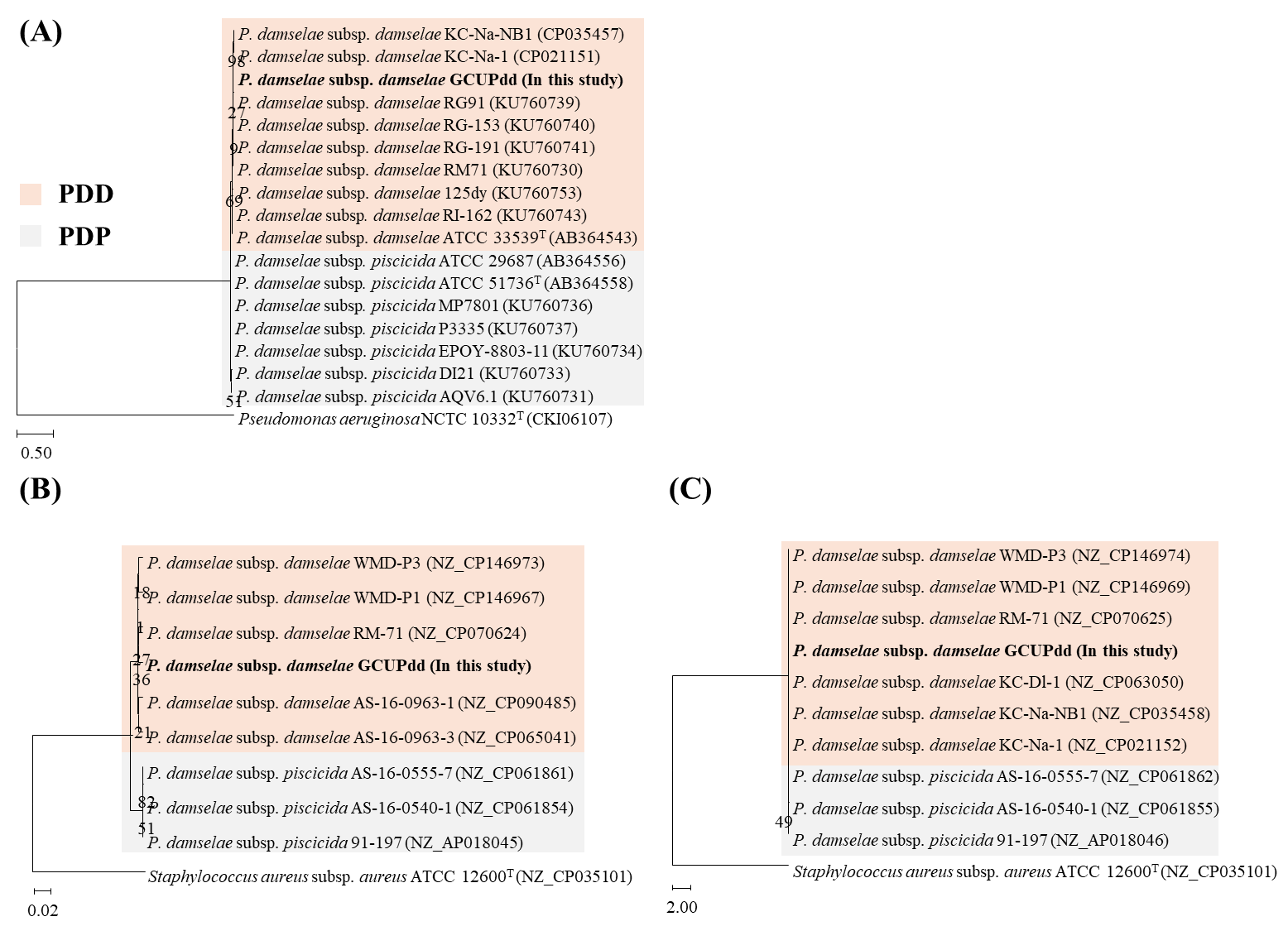


Figure S3: Phylogenetic analyses of the PDD strain GCUPdd based on the single gene (A) and genome (B and C) against the representative PDD and PDP strains available in the GenBank database. Maximum-likelihood phylogeny based on the amino acid sequences of the *toxR* gene (A) was reconstructed using MEGAX (v10.0). Whole-genome-based phylogenetic trees were constructed using the Type (Strain) Genome Server (TYGS) based on chromosome I (B) and chromosome II (C). The colored boxes represent members of PDD (pink) and PDP (grey). The outgroups are *Pseudomonas aeruginosa* NCTC 10332^T^ (A) and *Staphylococcus aureus* ATCC 12600^T^ (B and C), respectively.

# Table S1: Biochemical test results for the PDD strain GCUPdd using the API 20E kit.

| **Strain** | **Biochemical agents** | | | | | | | | | | | | | | | | | | | |
| --- | --- | --- | --- | --- | --- | --- | --- | --- | --- | --- | --- | --- | --- | --- | --- | --- | --- | --- | --- | --- |
|  | ONPG | ADH | LDC | ODC | CIT | H2S | URE | TDA | IND | VP | GEL | GLU | MAN | INO | SOR | RHA | SAC | MEL | AMY | ARA |
| GCUPdd | - | + | - | - | + | - | + | - | - | + | - | + | - | - | - | - | - | - | - | - |

β-galactosidase (ONPG), arginine dihydrolase (ADH), lysine decarboxylase (LDC), ornithine decarboxylase (ODC), citrate utilization (CIT), H2S production (H2S), urease (URE), tryptophane deaminase (TDA), indole production (IND), Voges-Proskauer (VP), gelatinase (GEL), glucose (GLU), mannitol (MAN), inositol (INO), sorbitol (SOR), rhamnose (RHA), saccharose (SAC), melibiose (MEL), amygdalin (AMY), and arabionse (ARA).

# Table S2: The putative virulence factors of the PDD strain GCUPdd identified using a Virulence Factor Database.

| **VFclass** | **Virulence factors** | **Related genes** | ***P.damselae* subsp. *damselae* GCUPdd (Prediction)** | | |
| --- | --- | --- | --- | --- | --- |
|  |  |  | **chromosome I** | **chromosome II** | **plasmid** |
| Adherence | Mannose-sensitive  hemagglutinin  (MSHA type IV pilus) | *mshA* | orf02432 | - | - |
|  |  | *mshE* | orf02436 | - | - |
|  |  | *mshG* | orf02435 | - | - |
|  |  | *mshH* | orf02443 | - | - |
|  |  | *mshL* | orf02439 | - | - |
|  |  | *mshM* | orf02438 | - | - |
|  | Type IV pilus | *pilA* | orf02356 | - | - |
|  |  | *pilB* | orf02357 | - | - |
|  |  | *pilC* | orf02358 | - | - |
|  |  | *pilD* | orf02359 | - | - |
| Chemotaxis and motility | Flagella | *cheA* | orf00779 | - | - |
|  |  | *cheB* | orf00780 | - | - |
|  |  | *cheR* | orf00737 | - | - |
|  |  | *cheV* | orf00736 | - | - |
|  |  | *cheW* | orf00783 | - | - |
|  |  | *cheY* | orf00777 | - | - |
|  |  | *cheZ* | orf00778 | - | - |
|  |  | *filM* | orf00766 | - | - |
|  |  | *flaE* | orf00750 | - | - |
|  |  | *flgB* | orf00738 | - | - |
|  |  | *flgC* | orf00739 | - | - |
|  |  | *flgD* | orf00740 | - | - |
|  |  | *flgE* | orf00741 | - | - |
|  |  | *flgF* | orf00742 | - | - |
|  |  | *flgG* | orf00743 | - | - |
|  |  | *flgH* | orf00744 | - | - |
|  |  | *flgI* | orf00745 | - | - |
|  |  | *flgJ* | orf00746 | - | - |
|  |  | *flhA* | orf00773 | - | - |
|  |  | *flhB* | orf00772 | - | - |
|  |  | *flhF* | orf00774 | - | - |
|  |  | *flhG* | orf00775 | - | - |
|  |  | *fliA* | orf00776 | - | - |
|  |  | *fliE* | orf00758 | - | - |
|  |  | *fliF* | orf00759 | - | - |
|  |  | *fliG* | orf00760 | - | - |
|  |  | *fliI* | orf00762 | - | - |
|  |  | *fliJ* | orf00763 | - | - |
|  |  | *fliL* | orf00765 | - | - |
|  |  | *fliN* | orf00767 | - | - |
|  |  | *fliO* | orf00768 | - | - |
|  |  | *fliP* | orf00769 | - | - |
|  |  | *fliQ* | orf00770 | - | - |
|  |  | *fliR* | orf00771 | - | - |
|  |  | *fliS* | orf00754 | - | - |
|  |  | *flrA* | orf00755 | - | - |
|  |  | *flrB* | orf00756 | - | - |
|  |  | *flrC* | orf00757 | - | - |
|  |  | *motA* | orf00669 | - | - |
|  |  | *motB* | orf00670 | - | - |
|  |  | *motX* | orf00254 | - | - |
|  |  | *motY* | orf01884 | - | - |
| Secretion system | EPS type II  secretion system | *epsE* | orf00144 | - | - |
|  |  | *epsF* | orf00145 | - | - |
|  |  | *epsG* | orf00146 | - | - |
|  |  | *epsI* | orf00148 | - | - |
|  |  | *epsJ* | orf00149 | - | - |
|  |  | *epsK* | orf00150 | - | - |
|  |  | *epsM* | orf00152 | - | - |
|  |  | *gspD* | orf00143 | - | - |
|  | VAS effector  proteins | *hcp-2* | orf01302 | orf03345 | orf03869 |
|  |  | *vgrG-2* | - | orf03344 | - |
|  | VAS type VI  secretion system | *vasA* | orf01283 | - | - |
|  |  | *vasB* | orf01284 | - | - |
|  |  | *vasD* | orf01286 | - | - |
|  |  | *vasE* | orf01287 | - | - |
|  |  | *vasF* | orf01288 | - | - |
|  |  | *vasG* | orf01289 | - | - |
|  |  | *vasK* | orf01293 | - | - |
| Toxin | Thermolabile  hemolysin | *tlh* | orf01185 | - | - |
| Immune evasion | Capsule  (*Streptococcus*) | *neuB* | orf01993 | - | - |
|  | Exopolysaccharide  (*Haemophilus*) | *galU* | orf01958; orf01981 | - | - |

# Table S3: Antimicrobial resistance gene screening results of the PDD strain GCUPdd using a Comprehensive Antibiotic Resistance Database.

| **RGI Criteria** | **Location (bp)** | **ARO Term** | **SNP** | **Detection Criteria** | **AMR Gene Family** | **Drug Class** | **Resistance Mechanism** | **% Identity of Matching Region** | **% Length of Reference Sequence** |
| --- | --- | --- | --- | --- | --- | --- | --- | --- | --- |
| **Chromosome Ⅰ** | | | | | | | | | |
| Strict | 214293-215477 | *Escherichia coli* EF-Tu mutants conferring resistance to Pulvomycin | R234F | Protein variant model | Elfamycin resistant EF-Tu | Elfamycin antibiotic | Antibiotic target alteration | 86.51 | 96.33 |
| Strict | 1262199-1262552 | qacJ |  | Protein homolog model | Small multidrug resistance (SMR) antibiotic efflux pump | Disinfecting agents and antiseptics | Antibiotic efflux | 40 | 109.35 |
| Strict | 2499454-2502564 | adeF |  | Protein homolog model | Resistance-nodulation-cell division (RND) antibiotic efflux pump | Fluoroquinolone antibiotic, tetracycline antibiotic | Antibiotic efflux | 42.2 | 97.83 |
| Strict | 2802362-2804092 | *Haemophilus influenzae* PBP3 conferring resistance to beta-lactam antibiotics | D350N, S385T | Protein variant model | Penicillin-binding protein mutations conferring resistance to beta-lactam antibiotics | Cephalosporin, cephamycin, penam | Antibiotic target alteration | 48.33 | 94.43 |
| Strict | 2926375-2927559 | *Escherichia coli* EF-Tu mutants conferring resistance to Pulvomycin | R234F | Protein variant model | Elfamycin resistant EF-Tu | Elfamycin antibiotic | Antibiotic target alteration | 86.77 | 96.33 |
| Strict | 2944000-2944632 | CRP |  | Protein homolog model | Resistance-nodulation-cell division (RND) antibiotic efflux pump | Macrolide antibiotic, fluoroquinolone antibiotic, penam | Antibiotic efflux | 95.71 | 100 |
| **Chromosome Ⅱ** | | | | | | | | | |
| Strict | 696622-699777 | adeF |  | Protein homolog model | Resistance-nodulation-cell division (RND) antibiotic efflux pump | Fluoroquinolone antibiotic, tetracycline antibiotic | Antibiotic efflux | 43.41 | 99.24 |
